# Stage Children

**Published:** 1920-01-31

**Authors:** 


### STAGE CHILDREN.

It is good to find that the authorities are taking steps to exercise a close supervision of children who are on the stage. Actors and actresses are notably kind people, and there is no reason to suppose that theatrical managers are more deficient in the humanities than any other section of the nation's employers. But there are many opportunities for abuse by unscrupulous people, and it is well that every care should be taken to safeguard the interests and the future of talented young boys and girls.

The Board of Education has issued an order having for its purpose a more intimate overseership by local authorities in the granting of licences for children taking part in theatrical and music-hall performances. As a general principle, one might deplore the appearance of small children on the public stage, but the fact exists and, be it said, gives much charm to the stage. Therefore, in those circumstances, the best must be done to look after their welfare. The Board of Education lays down certain broad principles to be observed in guaranteeing adequate holidays, and it is assumed that the persons dealing with the application will insist on seeing the child for whom a licence is desired. Questions addressed to the child itself will afford one of the best means of determining the propriety of granting a licence. Great importance is attached to the condition that the matron, governess, or other fit person who is to be responsible for the welfare of the children must be approved by the local education authority. With a view to assisting authorities in the exercise of their functions, the Board propose themselves to take steps for establishing a list of persons whom they regard as suitable for employment as matrons. Applications for enrolment on the list will be invited, and it will be open to local authorities to forward recommendations. The applicants will be given an opportunity of appearing before a committee which will be appointed by the Board.

Attention is called to the importance of making inquiries as to the earnings which the children will receive, and also as to the proposed manner of dealing with those earnings, with particular reference to the undesirability of allowing children to be exploited for the benefit of others. It would be desirable where possible to secure from applicants for licences an assurance that some part at any rate of the earnings of the children will be placed in a bank or otherwise preserved for their future benefit.

The Board add that they have every reason to hope that the establishment of the new procedure and the present rules will considerably improve the conditions under which children are employed on the stage.

